# Combination of Maximum Shear Wave Elasticity Modulus and TIRADS Improves the Diagnostic Specificity in Characterizing Thyroid Nodules: A Retrospective Study

**DOI:** 10.1155/2018/4923050

**Published:** 2018-10-09

**Authors:** Jing Hang, Fan Li, Xiao-hui Qiao, Xin-hua Ye, Ao Li, Lian-fang Du

**Affiliations:** ^1^Department of Ultrasound, The First Affiliated Hospital of Nanjing Medical University, Nanjing 210029, China; ^2^Department of Ultrasound, Shanghai General Hospital of Nanjing Medical University, Shanghai 200080, China

## Abstract

**Objectives:**

The present study is aimed at evaluating the diagnostic value of combining shear wave elastography (SWE) parameters and the thyroid imaging reporting and data system (TIRADS) for differentiating between benign and malignant thyroid nodules.

**Methods:**

Patients who underwent conventional ultrasonography (US) and SWE before surgery were enrolled in the current study. Each nodule was given a TIRADS risk score. The effectiveness of the SWE parameters was assessed by odds ratios (ORs). The SWE scoring risk stratification was proposed beyond 95% probability, and the desired values were obtained according to the log-normal distribution. The area under the receiver-operating characteristic (AUC) was used to compare the diagnostic performance between TIRADS-alone and TIRADS + SWE.

**Results:**

A total of 262 patients with 298 thyroid nodules were enrolled in our study. The pathological analyses were conducted on 121 benign and 177 malignant nodules. The AUC values for TIRADS-alone and TIRADS + SWE were 0.896 (accuracy 83.2%) and 0.917 (accuracy 84.2%), respectively. However, the TIRADS + SWE scores showed a higher specificity (88.4%) and positive predictive value (91.2%) as compared with the TIRADS-alone of 73.6% and 83.2%, respectively.

**Conclusions:**

Combining SWE and TIRADS improves the specificity of TIRADS-alone in differentiating between benign and malignant thyroid nodules.

## 1. Introduction

Despite the high prevalence of thyroid nodules in older patients, less than 10% of thyroid nodules are malignant at the time of diagnosis, regardless of whether the detected lesions are solitary or in a multinodular goiter [1, 2]. Since only 2–6% of thyroid nodules are palpable, high-frequency ultrasonography (US) has become the standard technique for detecting thyroid nodules. Currently, 68% of patients who undergo US have thyroid nodules [3]. For these reasons, all patients with suspected or confirmed thyroid nodules are recommended to undergo thyroid US [4]. According to the 2015 American Thyroid Association Management Guidelines, the following signs increase the risk of malignancy, including irregular margins, taller-than-wider shape, microcalcification, and marked hypoechogenicity [4]. However, using each characteristic sign individually to predict malignancy has a relatively low sensitivity (26.5–59.1%) [4–7]. Furthermore, while conventional US is extremely sensitive in detecting thyroid nodules, it cannot reliably differentiate the few malignant nodules [8].

Most malignant thyroid nodules are associated with increased nodular stiffness and are harder than the majority of benign nodules [9]. As a new tool to measure the tissue stiffness, US elastography has been utilized in assessing the thyroid cancer among thyroid nodules [10]. Shear wave elastography (SWE) can supplement conventional US by allowing for the quantitative evaluation of tissue hardness. The elasticity index (EI) provides quantitative information about SWE (expressed in m/s) and the estimated tissue stiffness (expressed in kilopascals (kPa)) [11]. The modulus of elasticity, also known as Young's modulus (in kPa), is calculated based on the SWE. The Young's modulus increases as the tissue becomes harder [12, 13]. A previous study reported that SWE is not superior to conventional US for diagnosing malignant thyroid disease [14]. In a previous large-scale study, the mean elasticity index (EI) values (*E*_mean_) and maximum EI value (*E*_max_) were found to be independent predictors of malignancy and the combination of SWE with gray-scale US allowed for improved prediction of thyroid malignancy [15].

Recently, several US-based systematic classification schemes have been developed that predict malignancy based on the US features of thyroid nodules [16]. Currently, the Kwak TIRADS is considered to be the most valuable tool for assessing routine thyroid US [17, 18]. Since the ACR-BIRADS has been widely used in breast imaging, the American College of Radiology developed the ACR-TIRADS in 2017 [19], which is the most up-to-date edition at this time. To the best of our knowledge, limited studies have investigated how the combination of SWE parameters with TIRADS categories affects the sensitivity of thyroid nodule detection and differentiation [20]. The aim of the present study was to determine whether combining TIRADS and SWE parameters aids in the discrimination of benign and malignant thyroid nodules.

## 2. Materials and Methods

The institutional review board of the Shanghai General Hospital of Nanjing Medical University reviewed and approved this retrospective study. The data were used as standard clinical protocol according to the ethical guidelines of the Helsinki Declaration.

### 2.1. Patients

From August 2016 to November 2017, a total of 262 patients with 298 thyroid nodules who underwent conventional US and SWE examination before surgery, were enrolled in this retrospective study. The surgical patients included those who received nondiagnostic or indeterminate results from a US-guided FNA biopsy, malignancy or suspected malignancy based on US-guided FNA results, or compressive symptoms. We only analyzed the patients who had completed SWE and conventional US imaging. The inclusion criteria for the patients were as follows: (a) age > 18 years; (b) no therapy or biopsy before US examination; (c) diameter of the thyroid nodule between 5 and 30 mm; (d) patients with solid or mostly solid thyroid nodules as assessed by US examination (cystic part < 50%); and (e) distance from the skin surface to nodular center was <25 mm, as this could be fully included in the maximum range of the SWE color overlay.

### 2.2. US Examination

Thyroid US imaging was performed using a real-time US device (Aixplorer, SuperSonic Imagine, Aix-en-Provence, France) equipped with a 4–15 MHz linear array transducer. The same transducer was also used for the SWE imaging. The patients underwent grey-scale color US and SWE examinations conducted by two different operators who had more than three years of thyroid US experience. The operators were blinded to the clinical data.

All the parameters were adjusted to allow for distinct and complete imaging of the nodules. SWE images were obtained for thyroid nodules in the longitudinal plane after conventional US. The transducer was held without compression or movement when SWE was activated. The real-time elastograms were displayed in dual mode alongside the grey-scale US images for evaluating the anatomical location. A square region-of-interest (ROI) was drawn to encompass the entire nodule and the surrounding tissues. On SWE mappings, a default chromatic scale with succession from blue to red was representative of soft-to-hard tissue stiffness. The tissue elastic modulus was expressed in kPa, and an upper limit of the display was set to 100 kPa. We acquired three or more SWE cine loops that lasted for >10 seconds (s) from each lesion for analysis. During the SWE examinations, all patients were asked to hold their breath and refrain from other movements for at least 3 s.

### 2.3. Image Analysis

Two doctors with more than three years of experience in thyroid US analyzed the images. According to TIRADS lexicon [21], a careful evaluation was performed to obtain the following grey-scale features: composition (solid, predominately solid, predominately cystic, and cystic), echogenicity (very hypoechoic as compared to the strap muscles; hypoechoic, isoechoic, or hyperechoic as compared to the adjacent thyroid parenchyma), taller-than-wider shape (measured in the transverse plane), nodule size, margin (smooth, irregular, ill defined, and lobulated), and presence of echogenic foci, peripheral calcifications, and comet-tail artifacts. Each nodule was assigned a TIRADS score based on the image characteristics [19].

**Figure 1:**
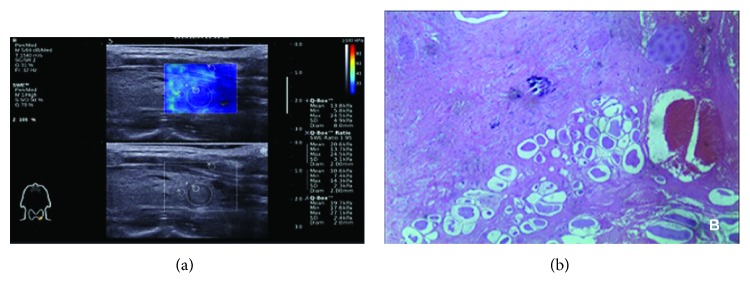
Thyroid follicular adenoma in a 52-year-old woman. (a) Conventional US image represents isoechoic, well-defined margins and without microcalcification. 2D-SWE mapping shows a homogeneous blue pattern indicative of a low Young's modulus. (b) Histological imaging was performed for verification after surgery; HE, 40x magnification.

**Figure 2:**
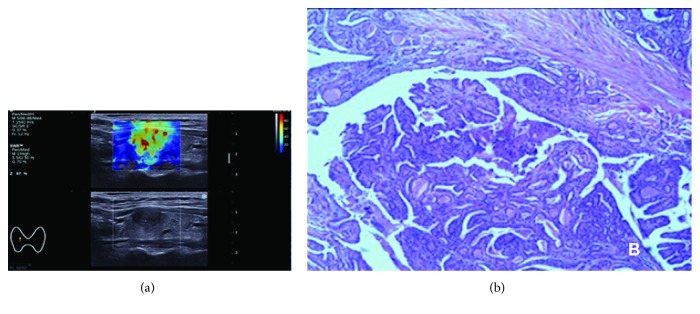
Thyroid papillary carcinoma in a 56-year-old woman. (a) Conventional US image represents a very hypoechoic, solid, and extrathyroidal extension. 2D-SWE mapping shows yellow and red areas indicative of a high Young's modulus. (b) Histological imaging was performed for verification after surgery; HE, 40x magnification.

The same two doctors analyzed the SWE images. The SWE measurements were performed by the Aixplorer, developed by SuperSonic Imagine (Aixplorer, SuperSonic Imagine, Aix-en-Provence, France). A circular ROI (i.e., Q-box: SuperSonic Imagine) was drawn around the thyroid nodule using the US images. Next, the *E*_mean_ and *E*_max_ were measured. For *E*_max_, a fixed 2 × 2 mm ROI was placed on the hardest part of the nodule, excluding calcifications. In addition, 2 × 2 mm ROIs were placed on the same level of the adjacent strap muscle to calculate the mean (SWE-mean-muscle) and maximum (SWE-max-muscle) EI values (Figures 1 and 2). *E*_ratio_ was calculated as the *E*_max_ divided by SWE-max-muscle. Each parameter was estimated in triplicate.

### 2.4. Pathological Examination

The pathological results were acquired by two pathologists with more than five years of experience in thyroid pathology. Nodules were classified as benign or malignant based on the postoperative pathological findings.

### 2.5. Statistical Analysis

The first model contained standard US features and three SWE parameters (*E*_mean_, *E*_max_, and *E*_ratio_). The second model was a probability model. The highest SWE parameter was counted according to a normal distribution. The log-normal distribution models for two groups were performed based on the pathological results. Risk stratification was proposed beyond 95% probability and expected values were calculated based on the log-normal distribution. Each nodule was assigned an SWE score based on the stratification, and the sum of the SWE and TIRADS scores was considered to be the final score for each nodule. The final model contained the TIRADS score and the sum of the two stratifications.

All of the parameters were described as ±S, and the two-tailed *p* < 0.05 was considered to be statistically significant. The odds ratios (ORs) and 95% confidence intervals (CIs) were calculated. Risk-stratification scoring was proposed as beyond 95% probability, and the desired values were obtained according to the log-normal distribution. The area under the ROC curve (AUC) was used to compare the diagnostic performance. The statistical analyses were performed using SPSS 19.0 (IBM Corporation, Armonk, NY, USA), MedCalc 12.2.0.0 software (MedCalc Software, Mariakerke, Belgium), and MATLAB 7.0 (MathWorks Corporation, Natick, MA, USA).

## 3. Results

### 3.1. Patients and Histological Results

Based on the inclusion criteria, 63 patients with 71 nodules were excluded from this study. In total, 262 patients with 298 thyroid nodules were recruited. The final cohort was comprised of 51 males and 247 females. The patients were found to have one, two, or three thyroid nodules in 88.2% (*n* = 231), 9.5% (*n* = 25), and 2.3% (*n* = 6) of the patients, respectively. The mean age of the patients was 45.57 ± 12.11 (range, 21–76) years. The mean diameter of the tumor nodules was 12.8 ± 6.3 (range 5.0–27.8) mm. All of the pathological results are summarized in Table 1.

### 3.2. TIRADS and SWE Results

According to the categorical variables, the US features of each nodule were compared using the chi-square test or Fisher's exact test. All of the variables were found to be significantly different between the two groups (*p* < 0.001).  Table 2 shows the US features between benign and malignant nodules. The risk value of each nodule was assigned based on the US feature, and the total score was calculated according to the ACR-TIRADS Committee [19].  Table 3 shows the malignancy rate based on the TIRADS score. The SWE parameters were compared by using an independent sample *t*-test (Table 4). Logistic analysis revealed that *E*_max_ had the highest ORs for malignancy (OR: 1.520, *p* < 0.001).

**Figure 3:**
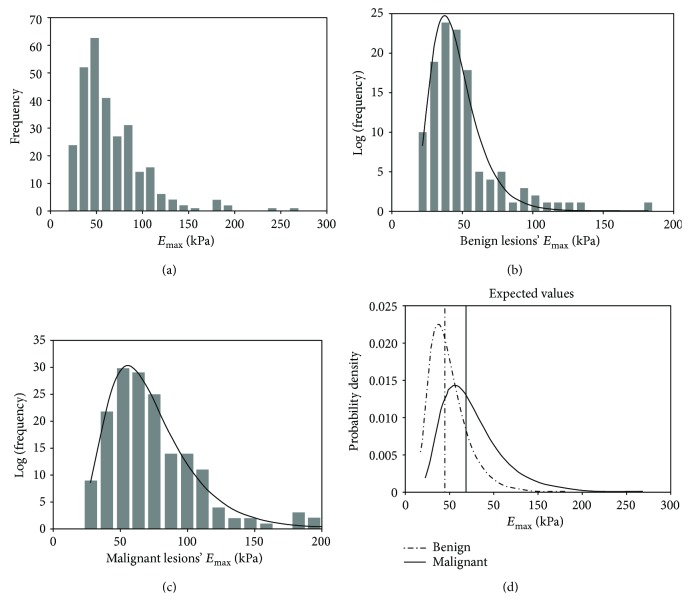
Statistical analysis curve of *E*_max_. (a) Normal frequency distribution histogram on *E*_max_. (b) Log-normal frequency distribution curve on *E*_max_ of benign lesions. (c) Log-normal frequency distribution curve on *E*_max_ of malignant lesions. (d) Probability density function curve and the expected values for benign and malignant nodules based on the logarithmic normal distribution.

*E*_max_ was found to be the most effective parameter in differentiating benign and malignant thyroid nodules and an *E*_max_ of 52.7 kPa was found to be the cutoff value from the current study. As TIRADS + SWE, a supplemental value related to the level of malignancy was obtained from the probability statistics of *E*_max_. We first drew normal frequency distribution histograms on *E*_max_ (Figure 3(a)) and found that this model was not exactly normally distributed. Next, we created the log-normal frequency distribution curve based on pathological results of the two groups (Figures 3(b) and 3(c)). Lastly, we calculated the desired value and variance yields of each group (Figure 3(d)). The log-desired value and variance value for benign nodules were 3.812 and 0.4297, respectively. This log-desired value corresponded to an *E*_max_ of 45.2 kPa. The log-desired value and variance value for the malignant nodules were 4.225 and 0.4465, respectively. This log-desired value corresponded to an *E*_max_ of 69.1 kPa. Hence, 45.2 kPa and 69.1 kPa were regarded as the desired values for benign and malignant groups, respectively. All of the statistical results are assimilated in Table 5.

### 3.3. Identification of the Boundary Range Based on the *E*_max_ Value

According to the expected and beyond 95% probability value, we presented a distribution range of *E*_max_ values in differentiating the thyroid nodules. The nodule was considered to be highly suspected for malignancy if the *E*_max_ was ≥120 kPa. The nodule was suspected of malignancy if the *E*_max_ was between 69 and 120 kPa. An *E*_max_ of 69 kPa was considered as the cutoff value for expected malignant nodules, while nodules between 45 and 69 kPa were considered indeterminate. If the *E*_max_ was less than 45 kPa, the nodule was suspected to be benign with the possibility of malignancy being <50%. The statistical and pathological results are summarized in Table 6.

### 3.4. Combination of SWE and TIRADS Points

According to the distribution range and malignancy rate, each nodule was assigned an SWE score from one to four with higher scores indicative of increased stiffness and higher malignancy suspicion (Table 6). The SWE and TIRADS points for each nodule were then combined, as shown in Table 7.

**Figure 4:**
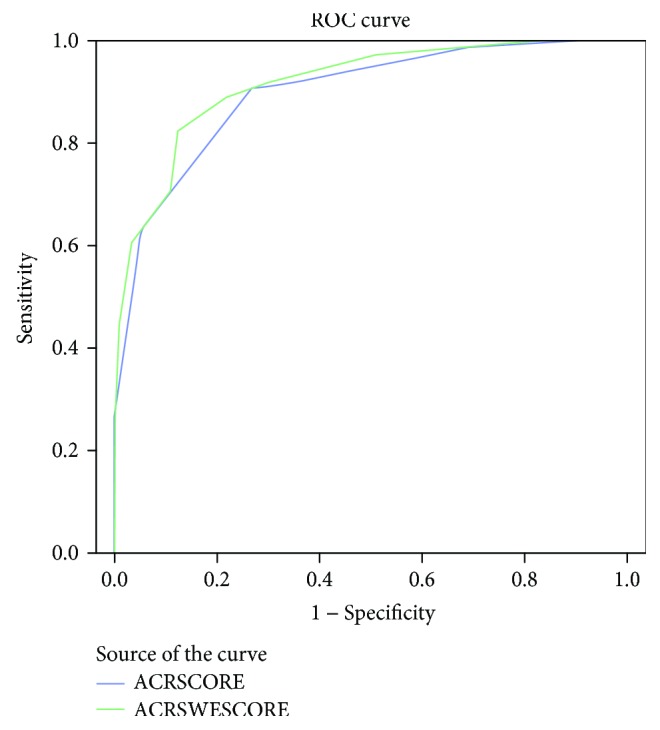
ROC curves of TIRADS and SWE + TIRADS score risk.

We mapped the ROC curves for two risk score (Figure 4) models, and the AUC values for each category were obtained based on the cutoff value for the comparison of sensitivity and specificity. The AUC and accuracy were very similar with and without SWE. However, the TIRADS + SWE combined scores showed a higher specificity (88.4%) and positive predictive value (91.2%) as compared to the TIRADS score (Table 8).

## 4. Discussion

US for imaging thyroid nodules was previously recommended in the 2015 American Thyroid Association Management Guidelines [4]. US has been widely used in patients with known and suspected thyroid nodules. In combination with US, some investigators have adopted “score-based” approaches in malignancy risk stratification [22, 23]. Due to the convenience of this routine work, clinicians tended to use this method for evaluating the malignancy potential of thyroid tumors. Furthermore, Middleton et al. conducted a multi-institutional analysis, which reported malignancy rates of <2% for TIRADS1 and TIRADS2, 5% for TIRADS3, 5–20% for TIRADS4, and >20% for TIRADS5 [24]. In the current study, the malignancy rate was higher for TIRADS3 and TIRADS4 as compared with the study by Middleton et al. This difference may be attributed to the higher number of malignant tumors at 59.4% (177/298) in comparison with the number of benign nodules. Furthermore, the TIRADS risk score was suggested as an effective tool in clinical practice with an AUC of 0.896. The cutoff value was >5 points, and the sensitivity and specificity were found to be 89.8% and 73.6%, respectively.

The majority of malignant thyroid nodules are known as papillary thyroid carcinomas. US elastography, a recently developed and sophisticated imaging technique, has been used to evaluate tissue stiffness [25]. Real-time SWE imaging is an operator-independent, reproducible, and quantitative elastography technique [21, 26, 27]. The inter- and intraoperator reproducibility was found to be acceptable for quasistatic elastography with a correlation coefficient ranging from 0.73 to 0.79 for interobserver variability and 0.73 to 0.84 for intraobserver variability [27, 28].

Some studies have used SWE to assess the thyroid nodules in order to differentiate between benign and malignant tumors based on the EI cutoff value. A recent study by Park et al. [15] reported that a mean EI value ≥85.2 kPa and a maximum EI value ≥94 kPa were independent predictors of thyroid malignancy. Bhatia et al. [28] calculated the different cutoff values for EI using an ROI maintained at 2 mm. In addition, the increasing EI cutoff values (>10.3 kPa, >132 kPa) were found to be associated with increased specificity (8.9–100%) and decreased sensitivity (7.7–100%). The thresholds reported were different and the value span was relatively large. In the current study, *E*_max_ was considered to be maximally effective among the three SWE parameters (OR: 1.520), which is consistent with a previous study by Katarzyna et al. [29].

Previously, Katarzyna et al. [29] had reported the combination of SWE and B-mode parameters. This combination resulted in no significant improvements in the differentiation of malignant and benign thyroid nodules. However, these results were based on the combination of the SWE cutoff values and B-mode risk factors. In our current study, the AUC of the SWE + TIRADS score was 0.917 as compared to the TIRADS-alone score of 0.896, which showed no statistical difference in assessing malignant nodules. In addition, the accuracy of SWE + TIRADS was slightly higher than that of TIRADS-alone. Overall, SWE + TIRADS did not significantly improve the differentiation of thyroid nodules when compared with TIRADS-alone. Based on the cutoff value of >8, the sensitivity and specificity of the combination method were 81.9% and 88.4%, respectively. So, the specificity and PPV values were higher for the combination of SWE + TIRADS compared with TIRADS-alone. Since most benign thyroid nodules and some small malignant lesions show indolent and nonprogressive behavior, not all of the known or suspicious nodules need to be biopsied or excised. A recent report showed that partial resections still played a role during benign goiter surgery [30]. Some studies suggested a patient-tailored and less aggressive multimodal therapeutic approach in the treatment of differentiated thyroid cancer (DTC) [31], and a low locoregional recurrence rate may be observed after total thyroidectomy without prophylactic lymph node dissection [32]. In addition, the role of routine neck dissection in the treatment of clinically node-negative PTC patients is currently being investigated [33]. In our study, we aimed to discover an improved approach for differentiating between malignant and benign thyroid nodules with high specificity. According to the current results, the SWE + TIRADS score showed improved specificity as compared with the TIRADS-alone score, suggesting that the combination method may be valuable in reducing unnecessary FNA biopsies in some patients.

## 5. Limitations

The present retrospective study had some limitations. First, the cases enrolled in the current study were surgical patients. We excluded the cases without surgical pathology. For this reason, the results may be inaccurate due to selection bias, suggesting that a prospective group control study is essential. Second, due to the insufficient sample size, a joint distribution function based on the *E*_max_ value could not be proposed. Additional information should be assimilated in future studies.

## 6. Conclusions

In summary, SWE imaging can provide quantitative information about the stiffness of thyroid nodules, despite some limitations. The quantitative parameters were effective in noninvasively differentiating between benign and malignant thyroid nodules. The TIRADS risk score was an effective clinical tool, and the combination of SWE and TIRADS scoring risk stratification improved the specificity in differentiating malignant thyroid nodules. This phenomenon might aid in reducing unnecessary FNA biopsies in some patients.

## Figures and Tables

**Table 1:** Pathological distribution of 298 thyroid nodules.

Pathological results	Number
*Malignant*	
Papillary carcinoma	175
Medullar carcinoma	2
Total	177
*Benign*	
Nodular goiter	45
Adenoma	68
Hashimoto's thyroiditis	5
Focal thyroiditis	3
Total	121
Overall total	298

**Table 2:** Conventional US features for differentiating thyroid lesions.

US features	Benign	Malignant	*P* value
Composition			
Cystic or spongiform	3	0	<0.001
Mixed	8	0	
Solid	110	177	
Echogenicity			<0.001
Anechoic	3	0	
Isoechoic or hyperechoic	46	18	
Hypoechoic	70	137	
Very hypoechoic	2	22	
Shape			<0.001
Wider than taller	115	138	
Taller than wider	6	39	
Margin			<0.001
Smooth or ill defined	95	35	
Lobulated or irregular	24	99	
Extrathyroidal extension	2	43	
Echogenic foci			<0.001
No echogenic foci	71	62	
Large comet-tail artifacts	29	2	
Macrocalcifications	8	5	
Peripheral (rim) calcifications	2	8	
Punctate echogenic foci	11	100	

**Table 3:** Malignancy rate for the TIRADS scoring system.

Risk score	Total	Benign	Malignant	Malignancy rate (%)
1	1	1	0	0
2	10	10	0	0
3	29	27	2	6.9
4	56	42	14	25.0
5	11	9	2	18.2
6	42	15	27	64.3
7	29	10	19	65.5
8	6	1	5	83.3
9	66	6	60	90.9
≥10	48	0	48	100
Total	298	121	177	59.4

**Table 4:** SWE parameters for differentiating thyroid lesions.

	Benign (kPa)	Malignant (kPa)	*P* value	Odds ratio	95% CI
*E*_mean_	22.5 ± 9.4	31.1 ± 10.5	0.049	1.014	0.961–1.069
*E*_max_	49.6 ± 25.5	78.7 ± 41.1	0.001	1.520	1.021–2.132
*E*_ratio_	2.68 ± 3.30	3.42 ± 2.08	0.742		

**Table 5:** Statistical results of *E*_max_ in benign and malignant thyroid nodules.

	No.	Distribution range (kPa)		Expected values		Variance yields *σ*
		Minimum	Maximum	Log (*x*)	*X* (kPa)	
Benign	121	18.0	177.8	3.812	45.2	0.4297
Malignant	177	22.8	278.5	4.225	69.1	0.4465

**Table 6:** Pathological distribution of 298 thyroid nodules based on *E*_max_ scores.

	*E*_max_ values				Total
	*E*_max_ < 45 kPa (1 point)	45 kPa ≤ *E*_max_ < 69 kPa (2 points)	69 kPa ≤ *E*_max_ < 120 kPa (3 points)	*E*_max_ ≥ 120 kPa (4 points)	
Benign	65	38	15	3	121
Malignant	26	61	71	19	177
Malignancy rate (%)	28.6	61.6	82.6	86.4	298

**Table 7:** Distribution of 298 thyroid lesions based on SWE + TIRADS scores.

Risk score	Benign	Malignant	Total	Malignancy rate (%)
2	1	0	1	0
3	6	0	6	0
4	13	0	13	0
5	41	6	47	12.8
6	21	8	29	27.6
7	14	7	21	33.3
8	11	11	22	50.0
9	4	21	25	84.0
10	6	17	23	73.9
11	3	31	34	91.2
12	1	37	38	97.4
≥13	0	39	39	100
Total	121	177	298	59.4

**Table 8:** Diagnostic performance of TIRADS and SWE + TIRADS scores.

	AUC	Sensitivity (%)	Specificity (%)	PPV (%)	NPV (%)	Accuracy (%)
TIRADS score (cutoff > 5)	0.896	89.8	73.6	83.2	83.2	83.2
SWE + TIRADS score (cutoff > 8)	0.917	81.9	88.4	91.2	77.0	84.2

## Data Availability

The data used to support the findings of this study are available from the corresponding author upon request.

## Abbreviations

TIRADS: Thyroid imaging reporting and data system
SWE: Shear wave elastography
US: Ultrasonography
ORs: Odds ratios
FNA: Fine-needle aspiration
ROIs: Region of interest
AUC: Area under the curve
EI: Elasticity index
CO: Confidence intervals.
